# Supplementary material for: Precision Medicine to Treat Urothelial Carcinoma—The Way Forward
Source: Cancers (Basel). 2023 Jun 1;15(11):3024. doi: 10.3390/cancers15113024 (PMC10252480; doi:10.3390/cancers15113024)
Supplement: Supplementary file 1 [file cancers-15-03024-s001.zip › cancers-2406374-supplementary.pdf]

Supplementary Materials: Precision Medicine to Treat Urothelial Carcinoma—The Way Forward

Carvy Floyd Luceno, Won Jin Jeon, Ravand Samaeekia, John Shin and Guru P. Sonpavde

Table S1. Clinical Trials with Results for Targeted Therapies for Urothelial Carcinoma (Monotherapy and Combination Therapies). [1–25]

Name	Target/ MOA	Trials	Phase, progress	*n*	Study arms	Primary endpoint(s)	Results
Pembrolizumab	PD-1	NCT02335424 (KEYNOTE-052)	II, completed	370	pembrolizumab as first-line therapy for locally advanced or metastatic cisplatin-ineligible UC	ORR	ORR: 28.6%, 95% CI 24.1-33.5
		NCT02256436 (KEYNOTE-045)	III, completed	542	pembrolizumab vs chemotherapy (investigator's choice of paclitaxel, docetaxel or vinflunine)	PFS and OS	PFS: 2.1 months (95% CI 2.0–2.2) vs 3.3 months (95% CI 2.4–3.6) (HR 0.96; 95% CI 0.79–1.16; *P*=0.31295)<br><br>OS: 10.1 months (95% CI 8.0–12.3) vs 7.3 months (95% CI 6.1–8.1) (HR: 0.70; 95% CI 0.57–0.85; *P*<0.001)<br><br>> 2 years follow up OS: 26.9% vs 14.3%<br><br>> 2 years follow up PFS: 12.4% vs 3.0%
		NCT01848834 (KEYNOTE-012)	Ib, completed	33	pembrolizumab (MK-3475) for advanced UC (Cohort C)	AEs, ORR	ORR: 26%, 95% CI 11–46
		NCT02500121	II, completed	108	pembrolizumab vs placebo	PFS	PFS: 5.4 months (95% CI, 3.1 to 7.3) vs 3.0 months (95% CI; 2.7 to 5.5) (HR 0.65, *p* = 0.04)
Pembrolizumab + Chemotherapy		NCT02853305 (KEYNOTE-361)	III, completed	1010	pembrolizumab with gemcitabine + cisplatin or carboplatin vs pembrolizumab or chemotherapy	PFS and OS	PFS: 8.3 months (95% CI 7.5–8.5) in P + chemotherapy vs 7.1 months (CI 6.4–7.9) in chemotherapy (HR: 0.78, 95% CI 0.65–0.93; p=0.0033)<br><br>OS: 17.0 vs 14.3 months (HR 0.86, 95% CI 0.72–1.02; p=0·0407)
Atezolizumab	PD-L1	NCT02108652 (IMvigor210 cohort B)	II, completed	119	atezolizumab in cisplatin-ineligible mUC	ORR	ORR: 23% (95% CI 16 to 31)

		NCT02302807 (IMvigor211)	III, completed	931	atezolizumab vs chemotherapy (investigator's choice of vinflunine, paclitaxel, or docetaxel)	OS	OS: 11.1 months (95% CI 8.6-15.5) vs 10.6 months (95% CI 8.4-12.2) (HR 0.87, 95% CI 0.63-1.21; p=0.41)
		NCT02807636 (IMvigor130)	III, active, not recruiting	1213	Group A: atezolizumab + platinum-based chemotherapy (gemcitabine plus either carboplatin or cisplatin)<br>Group B: atezolizumab monotherapy<br>Group C: placebo + platinum-based chemotherapy	OS, PFS, and AEs	OS: Group A 16.0 months (95% CI 13.9-18.9) vs Group B 15.7 months (95% CI 13.1-17.8) vs Group C 13.4 months (95% CI 12-15.2)<br><br>PFS: Group A 8.2 months (95% CI 6.5-8.3) vs Group C 6.3 months (95% CI 6.2-7.0) (HR 0.82, 95% CI 0.70-0.96, p=0.007)
Atezolizumab + Rogaratinib	PD-L1 and FGFR	NCT03473756 (FORT-2)	Ib/II, active, not recruiting	26	Rogaratinib + atezolizumab in cisplatin-ineligible advanced/ mUC	DLTs, TEAEs	TEAEs: diarrhea, hyperphosphatemia, nausea. Favorable safety profile.
Nivolumab	PD-1	NCT02387996 (CheckMate275)	II, completed	265	Nivolumab as a second-line for mUC	ORR	ORR: 19.6% (95% CI 15.0-24.9)
		NCT02632409 (CheckMate274)	III, active, not recruiting	709	nivolumab vs placebo in muscle-invasive urothelial carcinoma after radical cystectomy	DFS	DFS: 20.8 months (95% CI 15.6-27.6) vs 10.8 months (95% CI 8.3-13.9)
Nivolumab + Ipilimumab		NCT01928394 (CheckMate032)	I/II, active, not recruiting	274	Nivolumab monotherapy every 2 weeks (NIVO3) vs nivolumab 3 mg/kg plus ipilimumab 1 mg/kg every 3 weeks for four doses followed by nivolumab monotherapy 3 mg/kg every 2 weeks (NIVO3+IPI1)vs nivolumab 1 mg/kg plus ipilimumab 3 mg/kg every 3 weeks for four doses followed by nivolumab monotherapy 3 mg/kg every 2 weeks (NIVO1+IPI3)	ORR	ORR:<br>NIVO3 arm: 25.6% (95% CI, 16.4-36.8)<br>NIVO3+IPI1 arm: 26.9% (95% CI, 18.7-36.5%)<br>NIVO1+IPI3 arm: 38% (95% CI, 28.1-48.8)
Nivolumab + NKTR-214 (bempegaldesleukin)	PD-1 and interleukin-2 cytokine prodrug	NCT02983045 (PIVOT-02)	I/II, completed	34	NKTR-214 + nivolumab in advanced/ mUC	ORR	ORR: 48% (95% CI 27–69)

Avelumab	PD-L1	NCT01772004 (JAVELIN Solid Tumor)	I, completed	249	Avelumab after first-line platinum-based chemotherapy	Best overall response	Best overall response (complete or partial response): 17% (95% CI 11–24)
		NCT02603432 (JAVELIN Bladder 100)	III, active, not recruiting	700	maintenance avelumab vs best supportive care (gemcitabine + cisplatin or carboplatin)	OS	OS: 23.8 months vs 15 months (HR 0.76, 95% CI 0.631-0.915, p=0.0036) PFS: 5.5 months vs 2.1 months (HR 0.54, 95% CI 0.457-0.645, p<0.0001)
Durvalumab	PD-L1	NCT01693562	I/II, completed	191	durvalumab in advanced/mUC with progression of disease or treatment-naive mUC	DLTs AEs, ORR	ORR: 17.8% (95% CI 12.7-24.0)
Durvalumab + Tremelimumab	PD-L1 and CTLA-4	NCT02516241 (DANUBE)	III, active, not recruiting	1032	durvalumab with or without tremelimumab (a CTLA-4 inhibitor) as first-line treatment for mUC vs chemotherapy	OS	OS: 14.4 months (95% CI 10·4–17.3) in the durvalumab monotherapy group, 15.1 months (13.1–18.0) in the durvalumab plus tremelimumab group, and 12.1 months (10.9–14.0) in the chemotherapy group (HR 0.85, 95% CI 0.72–1.02; p=0.075)
Cetrelimab + Erdafitinib	PD-1 and FGFR	NCT03473743 (NORSE)	Ib/II, active, not recruiting	125	Erdafitinib with or without cetrelimab	DLTs, ORR, AEs	Ib ORR: 52% II ORR: 68% in combination vs 33% in erdafitinib alone
Rogaratinib	FGFR	NCT03410693 (FORT-1)	II, III, completed	175	Rogaratinib vs chemotherapy in FGFR positive UC with disease progression during or after treatment with at least one platinum-containing regimen	ORR	ORR 20.7% (95% CI 12.7-30.7) vs 19.3% (95% CI 11.7-29.1)
Erdafitinib	FGFR	NCT02365597	II, active, not recruiting	99	Metastatic or surgically unresectable UC with disease progression despite chemotherapy	ORR	ORR: 40%
Enfortumab vedotin	Nectin-4 and MMAE						
		NCT03474107 (EV-301)	III, active, not recruiting	608	Enfortumab vedotin vs investigator's choice of chemotherapy (docetaxel, paclitaxel, or vinflunine)	OS	OS: 12.88 months vs 8.97 months (HR 0.70, 95% CI 0.56-0.89, p=0.001) PFS: 5.55 months vs 3.71 months (HR 0.62, 95% CI 0.51-0.75, p<0.001)

		NCT03219333 (Cohort 2 of EV-201)	II, active, not recruiting	89	Enfortumab vedotin for platinum-naive and cisplatin-ineligible advanced/ mUC	ORR	ORR: 52% (95% CI 41-62)
		NCT02091999 (EV-101)	I, active, not recruiting	112	Enfortumab vedotin as monotherapy for mUC	AEs, ORR	ORR: 42% (95% CI 31.2-52.5)
Enfortumab vedotin + pembrolizumab		NCT03288545 (EV-103)	III, active, recruiting	45	Previously untreated locally advanced/mUC	Safety	Manageable safety profile. ORR: 73.3% (95% CI, 58.1 to 85.4) OS: 26.1 months
Feladilimab (GSK3359609) + pembrolizumab + chemotherapy	Humanized IgG4 antibody with activity against T cell co-stimulator	NCT02723955 (INDUCE-1)	I, active, not recruiting	45	Feladilimab monotherapy vs fedladilimab + pembrolizumab	AEs, DLTs	ORR: 8% 95% CI: 0.2-36.0) in monotherapy vs 22% (95% CI 9.3-40) in combination therapy OS: 14.5 months (95% CI 2.8, NR) in monotherapy vs 10.7 months (95% CI: 5.2-18.1) in combination therapy
Autologous genetically modified MAGE-A4c1032 T cells	Adoptive T cell therapy	NCT03132922	I, active, not recruiting	60	HLA-A2+ patients with MAGE-A4 Positive Tumors	AE, DLT	ORR: 24% (95% confidence interval (CI): 11.4-40.2
Infilgratinib	FGFR	NCT04197986 (PROOF-302)	III, terminated	218	UC post resection	DFS	**Terminated**
Pemigatinib	FGFR	NCT04294277 (PEGASUS)	Terminated	2	Pemigatinib for UC who have received radical surgery	Relapse-free survival rate	**Terminated**

MOA: mechanism of action; UC: urothelial carcinoma; ORR: Objective Response Rate; CI: confidence interval; P: pembrolizumab; PFS: Progression Free Survival; OS: Overall Survival; HR: hazard ratio; PD-1: programmed cell death protein 1; AE: adverse event; PD-L1: programmed death ligand-1; mUC: metastatic urothelial carcinoma; FGFR: fibroblast growth factor receptor; DLT: dose-limiting toxicity; TEAE: treatment-emergent adverse events; DFS: Disease Free Survival; CTLA-4: cytotoxic T-lymphocyte-associated protein 4; MMAE: monomethyl auristatin E; NR: not reached

Table S2. Currently Ongoing Clinical Trials Pending Results for Targeted Therapies for Urothelial Carcinoma (Monotherapy).

Name	Target/ MOA	Trials	Phase	n	Study arms	Primary Endpoint(s)
Sacituzumab Govitecan	Anti-Trop-2 humanized monoclonal antibody + SN-38	NCT03547973 (TROPHY-U-01)	II, active, recruiting	643	Sacituzumab govitecan (6 cohorts)	ORR, PFS
Disitamab Vedotin	HER2 (hertuzumab and MMAE)	NCT04879329	II, active	270	Disitamab Vedotin monotherapy (only cohorts A and B) for HER2+ locally	Confirmed ORR

					advanced unresectable or mUC	
Pemigatinib	FGFR	NCT03914794	II, active, recruiting	43	high-risk UC post radical surgery	CR
4SCAR-PSMA 4SCAR-FRa	Adoptive T cell therapy	NCT03185468	I/II, active, recruiting	20	locally advanced or mUC with no further treatment available	OS, AEs
CCT301-59 CAR T	Adoptive T cell therapy	NCT03960060	I, active, not recruiting	18	recurrent or refractory solid tumors including bladder cancer	Safety, efficacy
TBI-1301 (NY-ESO-1 Specific TCR Gene Transduced Autologous T Lymphocytes)	Adoptive T cell therapy	NCT02869217	Ib, active, not recruiting	22	NY-ESO-1 expressing solid tumors in HLA-A2 positive patients (including BC)	Safety profile
HER2 specific CAR T cells + intra-tumor injection of CAdVEC	Adoptive T cell therapy	NCT03740256	I, active, recruiting	45	Advanced HER2 positive solid tumors including BC	DLTs

MOA: mechanism of action; ORR: Objective Response Rate; PFS: Progression Free Survival; HER2: human epidermal growth factor receptor 2; MMAE: monomethyl auristatin E; FGFR: fibroblast growth factor receptor; CR: Confirmed Response; OS: Overall Survival; AE: adverse event; mUC: metastatic urothelial carcinoma; UC: urothelial carcinoma; BC: bladder cancer; CAR: chimeric antigen receptor; DLT: dose-limiting toxicity

Table S3. Currently Ongoing Clinical Trials Pending Results for Targeted Therapies for Urothelial Carcinoma (Combination Therapy).

Name(s)	Target/ MOA	Trials	Phase	n	Study arms	Primary Endpoint(s)
Atezolizumab + CRT	PD-L1 + CRT	NCT03775265	III, active, recruiting	475	RT + chemotherapy vs CRT + atezolizumab in localized MIBC	Bladder intact event-free survival
Pembrolizumab + CRT	PD-1 + CRT	NCT04241185 (KEYNOTE-992)	III, active, recruiting	636	Pembrolizumab + CRT vs placebo + CRT in MIBC	Bladder intact event-free survival
Enfortumab vedotin + pembrolizumab + platinum-based chemotherapy	Nectin-4 and MMAE + PD-1	NCT03288545 (EV-103)	I/II, active, not recruiting	457	UC	ORR, pCR, AEs
Saciuzumab-govitecan + pembrolizumab	Anti-Trop-2 humanized monoclonal antibody + PD-1/PD-L1	NCT05535218 (SURE-02)	II, active, recruiting	48	MIBC	pCR
Sacituzumab-govitecan + Atezolizumab		NCT03869190 (MORPHEUS-UC)	Ib/II, active, recruiting	645	MIBC or locally advanced or mUC who progressed with platinum therapy	ORR, pCR
Sacituzumab-govitecan + Avelumab		NCT05327530 (JAVELIN Bladder Medley)	II, active, recruiting	252	MIBC or locally advanced or mUC who progressed with platinum therapy	PFS, AEs

Disitamab Vedotin + Pembrolizumab		NCT04879329	II, active	270	Disitamab Vedotin monotherapy (only cohort C) for HER2+ locally advanced unresectable or mUC	Confirmed ORR
Disitamab Vedotin + Toripalimab	HER2 (hertuzumab and MMAE) + PD-1/PD-L1	NCT05302284	III, active, recruiting	456	untreated unresectable locally advanced or metastatic HER2 positive UC	PFS, OS
RC48-ADC (Disitamab Vedotin) + Toripalimab		NCT04264936	Ib/II, active, unknown recruitment status	36	RC48-ACD and JS001 for locally advanced/mUC	AEs and maximal tolerated dose
EphB4-human serum albumin + pembrolizumab	EphB4-human serum albumin + PD-1	NCT02717156	II, active, recruiting	170	EphB4-HAS + pembrolizumab in solid tumors	Toxicities and AEs
Cabozantinib + avelumab (VEGF TKI + PD-L1 inhibitor)	VEGF TKI + PD-L1 inhibitor	NCT05092958 (MAIN-CAV)	III, active, recruiting	654	Avelumab vs avelumab + cabozantinib in mUC	OS

MOA: mechanism of action; CRT: chemoradiotherapy; PD-L1: programmed cell death ligand-1; RT: radiotherapy; PD-1: programmed cell death protein 1; MIBC: muscle invasive bladder cancer; MMAE: monomethyl auristatin E; UC: urothelial carcinoma; ORR: Objective Response Rate; pCR: pathological complete response; AE: adverse event; PFS: Progression Free Survival; mUC: metastatic urothelial carcinoma; OS: Overall Survival; HER2: human epidermal growth factor receptor 2; MMAE; ADC: antibody-drug conjugate; VEGF: vascular endothelial growth factor; TKI: tyrosine kinase inhibitor

References

1. Fradet, Y.; Bellmunt, J.; Vaughn, D.J.; Lee, J.L.; Fong, L.; Vogelzang, N.J.; Climent, M.A.; Petrylak, D.P.; Choueiri, T.K.; Necchi, A.; et al. Randomized phase III KEYNOTE-045 trial of pembrolizumab versus paclitaxel, docetaxel, or vinflunine in recurrent advanced urothelial cancer: Results of >2 years of follow-up. *Ann. Oncol.* **2019**, *30*, 970–976. https://doi.org/10.1093/annonc/mdz127.
2. Patel, M.R.; Ellerton, J.; Infante, J.R.; Agrawal, M.; Gordon, M.; Aljumaily, R.; Britten, C.D.; Dirix, L.; Lee, K.W.; Taylor, M.; et al. Avelumab in metastatic urothelial carcinoma after platinum failure (JAVELIN Solid Tumor): Pooled results from two expansion cohorts of an open-label, phase 1 trial. *Lancet Oncol.* **2018**, *19*, 51–64. https://doi.org/10.1016/s1470-2045(17)30900-2.
3. Powles, T.; Durán, I.; van der Heijden, M.S.; Loriot, Y.; Vogelzang, N.J.; De Giorgi, U.; Oudard, S.; Retz, M.M.; Castellano, D.; Bamias, A.; et al. Atezolizumab versus chemotherapy in patients with platinum-treated locally advanced or metastatic urothelial carcinoma (IMvigor211): A multicentre, open-label, phase 3 randomised controlled trial. *Lancet* **2018**, *391*, 748–757. https://doi.org/10.1016/s0140-6736(17)33297-x.
4. Loriot, Y.; Necchi, A.; Park, S.H.; Garcia-Donas, J.; Huddart, R.; Burgess, E.; Fleming, M.; Rezazadeh, A.; Mellado, B.; Varlamov, S.; et al. Erdafitinib in Locally Advanced or Metastatic Urothelial Carcinoma. *N. Engl. J. Med.* **2019**, *381*, 338–348. https://doi.org/10.1056/NEJMoa1817323.
5. Sternberg, C.N.; Petrylak, D.P.; Bellmunt, J.; Nishiyama, H.; Necchi, A.; Gurney, H.; Lee, J.-L.; van der Heijden, M.S.; Rosenbaum, E.; Penel, N.; et al. FORT-1: Phase II/III Study of Rogaratinib Versus Chemotherapy in Patients with Locally Advanced or Metastatic Urothelial Carcinoma Selected Based on FGFR1/3 mRNA Expression. *J. Clin. Oncol.* **2022**, *41*, 629–639. https://doi.org/10.1200/JCO.21.02303.
6. Powles, T.; Rosenberg, J.E.; Sonpavde, G.P.; Loriot, Y.; Durán, I.; Lee, J.L.; Matsubara, N.; Vulsteke, C.; Castellano, D.; Wu, C.; et al. Enfortumab Vedotin in Previously Treated Advanced Urothelial Carcinoma. *N. Engl. J. Med.* **2021**, *384*, 1125–1135. https://doi.org/10.1056/NEJMoa2035807.
7. Galsky, M.D.; Arija JÁ, A.; Bamias, A.; Davis, I.D.; De Santis, M.; Kikuchi, E.; Garcia-Del-Muro, X.; De Giorgi, U.; Mencinger, M.; Izumi, K.; et al. Atezolizumab with or without chemotherapy in metastatic urothelial cancer (IMvigor130): A multicentre, randomised, placebo-controlled phase 3 trial. *Lancet* **2020**, *395*, 1547–1557. https://doi.org/10.1016/s0140-6736(20)30230-0.
8. Powles, T.; Csőszi, T.; Özgüroğlu, M.; Matsubara, N.; Géczi, L.; Cheng, S.Y.S.; Fradet, Y.; Oudard, S.; Vulsteke, C.; Morales Barrera, R.; et al. Pembrolizumab alone or combined with chemotherapy versus chemotherapy as first-line therapy for advanced urothelial carcinoma (KEYNOTE-361): A randomised, open-label, phase 3 trial. *Lancet Oncol.* **2021**, *22*, 931–945. https://doi.org/10.1016/S1470-2045(21)00152-2.
9. Rosenberg, J.E.; Gajate, P.; Morales-Barrera, R.; Lee, J.-L.; Necchi, A.; Penel, N.; Zagonel, V.; Sierecki, M.R.; Piciu, A.-M.; Ellinghaus, P.; et al. Safety and preliminary efficacy of rogaratinib in combination with atezolizumab in a phase Ib/II study

(FORT-2) of first-line treatment in cisplatin-ineligible patients (pts) with locally advanced or metastatic urothelial cancer (UC) and FGFR mRNA overexpression. *J. Clin. Oncol.* **2020**, *38*, 5014–5014. https://doi.org/10.1200/JCO.2020.38.15_suppl.5014.

10. Siefker-Radtke, A.O.; Loriot, Y.; Siena, S.; Beato, C.; Duran, M.A.C.; Varlamov, S.; Duran, I.; Tagawa, S.T.; Geoffrois, L.; Mellado, B.; et al. 752P Updated data from the NORSE trial of erdafitinib (ERDA) plus cetrelimab (CET) in patients (pts) with metastatic or locally advanced urothelial carcinoma (mUC) and specific fibroblast growth factor receptor (FGFR) alterations. *Ann. Oncol.* **2020**, *31*, S584–S585. https://doi.org/10.1016/j.annonc.2020.08.824.
11. Sharma, P.; Siefker-Radtke, A.; de Braud, F.; Basso, U.; Calvo, E.; Bono, P.; Morse, M.A.; Ascierto, P.A.; Lopez-Martin, J.; Brossart, P.; et al. Nivolumab Alone and With Ipilimumab in Previously Treated Metastatic Urothelial Carcinoma: CheckMate 032 Nivolumab 1 mg/kg Plus Ipilimumab 3 mg/kg Expansion Cohort Results. *J. Clin. Oncol.* **2019**, *37*, 1608–1616. https://doi.org/10.1200/jco.19.00538.
12. Hoimes, C.J.; Flaig, T.W.; Milowsky, M.I.; Friedlander, T.W.; Bilen, M.A.; Gupta, S.; Srinivas, S.; Merchan, J.R.; McKay, R.R.; Petrylak, D.P.; et al. Enfortumab Vedotin Plus Pembrolizumab in Previously Untreated Advanced Urothelial Cancer. *J. Clin. Oncol.* **2022**, *41*, 22–31. https://doi.org/10.1200/JCO.22.01643.
13. Balar, A.V.; Moreno, V.; Angevin, E.; Gan, H.K.; Vieito, M.; Italiano, A.; Danielli, R.; Massarelli, E.; Opdam, F.; Chisamore, M.J.; et al. Inducible T-cell co-stimulatory (ICOS) receptor agonist, feladilimab (fela), alone and in combination (combo) with pembrolizumab (P): Results from INDUCE-1 urothelial carcinoma (UC) expansion cohorts (ECs). *J. Clin. Oncol.* **2021**, *39*, 4519–4519. https://doi.org/10.1200/JCO.2021.39.15_suppl.4519.
14. Vuky, J; Balar, A.V.; Castellano, D.; O'Donnell, P.H.; Grivas, P.; Bellmunt, J.; Powles, T.; Bajorin, D.; Hahn, N.M.; Savage, M.J.; et al. Long-Term Outcomes in KEYNOTE-052: Phase II Study Investigating First-Line Pembrolizumab in Cisplatin-Ineligible Patients With Locally Advanced or Metastatic Urothelial Cancer. *J Clin Oncol* **2020**, *38*, 2658-2666, doi:10.1200/jco.19.01213.
15. Plimack, E.R.; Bellmunt, J.; Gupta, S.; Berger, R.; Chow, L.Q.M.; Juco, J.; Lunceford, J.; Saraf, S.; Perini, R.F.; O'Donnell, P.H. Safety and activity of pembrolizumab in patients with locally advanced or metastatic urothelial cancer (KEYNOTE-012): a non-randomised, open-label, phase 1b study. *The Lancet Oncology* **2017**, *18*, 212-220, doi:10.1016/S1470-2045(17)30007-4.
16. Galsky, M.D.; Mortazavi, A.; Milowsky, M.I.; George, S.; Gupta, S.; Fleming, M.T.; Dang, L.H.; Geynisman, D.M.; Walling, R.; Alter, R.S.; et al. Randomized Double-Blind Phase II Study of Maintenance Pembrolizumab Versus Placebo After First-Line Chemotherapy in Patients With Metastatic Urothelial Cancer. *Journal of Clinical Oncology* **2020**, *38*, 1797-1806, doi:10.1200/JCO.19.03091.
17. Balar, A.V.; Galsky, M.D.; Rosenberg, J.E.; Powles, T.; Petrylak, D.P.; Bellmunt, J.; Loriot, Y.; Necchi, A.; Hoffman-Censits, J.; Perez-Gracia, J.L.; et al. Atezolizumab as first-line treatment in cisplatin-ineligible patients with locally advanced and metastatic urothelial carcinoma: a single-arm, multicentre, phase 2 trial. *The Lancet* **2017**, *389*, 67-76, doi:10.1016/S0140-6736(16)32455-2.
18. Galsky, M.D.; Retz, M.; Siefker-Radtke, A.O.; Baron, A.; Necchi, A.; Bedke, J.; Plimack, E.R.; Vaena, D.; Grimm, M.O.; Bracarda, S.; et al. Efficacy and safety of nivolumab monotherapy in patients with metastatic urothelial cancer (mUC) who have received prior treatment: Results from the phase II CheckMate 275 study. *Annals of Oncology* **2016**, *27*, vi567, doi:10.1093/annonc/mdw435.24.
19. Bajorin, D.F.; Witjes, J.A.; Gschwend, J.E.; Schenker, M.; Valderrama, B.P.; Tomita, Y.; Bamias, A.; Lebret, T.; Shariat, S.F.; Park, S.H.; et al. Adjuvant Nivolumab versus Placebo in Muscle-Invasive Urothelial Carcinoma. *New England Journal of Medicine* **2021**, *384*, 2102-2114, doi:10.1056/NEJMoa2034442.
20. Powles, T.; O'Donnell, P.H.; Massard, C.; Arkenau, H.T.; Friedlander, T.W.; Hoimes, C.J.; Lee, J.L.; Ong, M.; Sridhar, S.S.; Vogelzang, N.J.; et al. Efficacy and Safety of Durvalumab in Locally Advanced or Metastatic Urothelial Carcinoma: Updated Results From a Phase 1/2 Open-label Study. *JAMA Oncol* **2017**, *3*, e172411, doi:10.1001/jamaoncol.2017.2411.
21. Powles, T.; van der Heijden, M.S.; Castellano, D.; Galsky, M.D.; Loriot, Y.; Petrylak, D.P.; Ogawa, O.; Park, S.H.; Lee, J.-L.; De Giorgi, U.; et al. Durvalumab alone and durvalumab plus tremelimumab versus chemotherapy in previously untreated patients with unresectable, locally advanced or metastatic urothelial carcinoma (DANUBE): a randomised, open-label, multicentre, phase 3 trial. *The Lancet Oncology* **2020**, *21*, 1574-1588, doi:10.1016/S1470-2045(20)30541-6.
22. Powles, T.B.; Chistyakov, V.; Beliakouski, V.; Semenov, A.; Everaert, E.; Baranau, Y.; Moreno, V.; Perez Valderrama, B.; Vano, Y.; Del Conte, G.; et al. LBA27 Erdafitinib (ERDA) or ERDA plus cetrelimab (CET) for patients with metastatic or locally advanced urothelial carcinoma (mUC) and Fibroblast Growth Factor Receptor alterations (FGFRa): First phase (Ph) II results from the NORSE study. *Annals of Oncology* **2021**, *32*, S1303, doi:10.1016/j.annonc.2021.08.2103.
23. Yu, E.Y.; Petrylak, D.P.; O'Donnell, P.H.; Lee, J.-L.; van der Heijden, M.S.; Loriot, Y.; Stein, M.N.; Necchi, A.; Kojima, T.; Harrison, M.R.; et al. Enfortumab vedotin after PD-1 or PD-L1 inhibitors in cisplatin-ineligible patients with advanced urothelial carcinoma (EV‑201): a multicentre, single-arm, phase 2 trial. *The Lancet Oncology* **2021**, *22*, 872-882, doi:10.1016/S1470-2045(21)00094-2.
24. Rosenberg, J.E.; Sridhar, S.S.; Zhang, J.; Smith, D.C.; Ruether, J.D.; Flaig, T.W.; Baranda, J.C.; Lang, J.M.; Plimack, E.R.; Sangha, R.S.; et al. Mature results from EV-101: A phase I study of enfortumab vedotin in patients with metastatic urothelial cancer (mUC). *Journal of Clinical Oncology* **2019**, *37*, 377-377, doi:10.1200/JCO.2019.37.7_suppl.377.
25. Hong, D.S.; Van Tine, B.A.; Biswas, S.; McAlpine, C.; Johnson, M.L.; Olszanski, A.J.; Clarke, J.M.; Araujo, D.; Blumenschein, G.R.; Kebriaei, P.; et al. Autologous T cell therapy for MAGE-A4+ solid cancers in HLA-A*02+ patients: a phase 1 trial. *Nature Medicine* **2023**, *29*, 104-114, doi:10.1038/s41591-022-02128-z.
